# Antibacterial and Tribological Performance of Carbonitride Coatings Doped with W, Ti, Zr, or Cr Deposited on AISI 316L Stainless Steel

**DOI:** 10.3390/ma10101189

**Published:** 2017-10-17

**Authors:** Sun-Hui Yao, Yen-Liang Su, Yu-Cheng Lai

**Affiliations:** 1 Chang Jung Christian University, No. 1 Changda Road, Tainan 71101, Taiwan; 2 Department of Mechanical Engineering, National Cheng Kung University, No. 1 University Road, Tainan 70101, Taiwan; suyl@mail.ncku.edu.tw (Y.L.S.); n16054784@mail.ncku.edu.tw (Y.C.L.)

**Keywords:** carbon-nitride, metal doping, antibacterial performance, wear behavior

## Abstract

Carbonitride (CNx) coatings have existed for several decades but are not well understood. Related studies have indicated that CNx coatings exhibit behaviors comparable to diamond-like carbon (DLC) coatings. Metal-doped CNx coatings are expected to show superior performance to single CNx coatings. In this study, a CNx coating and a group of CNx coatings with 6 at. % metal doping (W, Ti, Zr, or Cr) were prepared on biograde AISI 316L stainless steel (SS316L) substrates, and they were then characterized and studied for antibacterial and wear performance. The microstructure, constituent phase, nanohardness, adhesion, surface roughness, and contact angle were evaluated. The antimicrobial test used *Staphylococcus aureus* and followed the Japanese Industrial Standard JIS Z 2801:2010. Finally, the wear behavior was assessed. The results showed that the CNx coating was a composite of amorphous CNx and amorphous C structures. The metal doping caused crystalline metal carbides/nitrides to form in the CNx coatings, which weakened their overall integrity. All the coatings showed antimicrobial ability for the SS316L samples. The CNx-Zr coating, the surface of which had the highest hydrophilicity, produced the best antibacterial performance. However, the CNx-Zr coating showed lower wear resistance than the CNx-W and CNx-Ti coatings. The CNx-Ti coating with a highly hydrophilic surface exhibited the lowest antibacterial ability.

## 1. Introduction

Considering a coating with antibacterial ability, the first candidate would be Ag-containing coating since Ag is a well-known antibacterial element. For example, the silver nanoclusters-silica composite coating with 0.4 at. **%** Ag showed antibacterial ability and improved bacterial anti-adhesion performance [1]. Another study on a magnetron-sputtered Zr-Cu-Ag composite coating found it showed good mechanical performance, high chemical inertness, and good antimicrobial ability [2]. 

In the literature, one can find some, but not much, researchers have endeavored to study the sputtered coatings against bacteria. Groessner-Schreiber et al. [3,4] found that the microflora and *Streptococcus* adhesion can be reduced by TiN and ZrN coatings. A study by Huang et al. [5] proved that the TaN coating possessed antibacterial ability against *Staphylococcus aureus* (*S. aureus*), with a conclusion due to the TaN coating being with high hydrophobic surface. In a study by Julius Andrew et al. [6], the magnetron-sputtered TiO_2_ coating on PMMA material was studied, which exhibited antibacterial efficiency reaching 70% and a large decrease in the contact angle from 33–66 degrees to 5–30 degrees.

Few studies have been conducted on applying carbon-based coatings against microbes; those which have were limited to diamond-like carbon (DLC) related coatings. Marciano et al. [7] evaluated the antibacterial ability of DLC coatings using *S. aureus*, *Escherichia coli* (*E. coli*), *Pseudomonas aeruginosa* (*P. aeruginosa*), and *Salmonella*. They found that a DLC coating with an H content of 22 at. % and an intensity ratio of D/G peaks (ID/IG) of 1.23 (detected by Raman spectroscopy) reduced bacterial activity by 25–55% compared with an uncoated sample, depending on the type of bacteria used. Zhou et al. [8] prepared DLC and hydrogenated DLC coatings on SS316L substrates and evaluated their antibacterial ability using *E. coli*. Compared with the uncoated sample, the DLC and hydrogenated DLC coatings reduced the viable bacteria to 15% and 33%, respectively. Studies on carbon-based coatings have demonstrated that surface properties are another factor affecting the adhesion of bacteria [9,10,11]. Ishihara et al. [12] prepared DLC coatings using a radio frequency magnetron sputtering system and evaluated the effect of the hydrophilicity of coating surfaces on the bacterial retention. The results indicated that the higher the hydrophilicity was, the less the bacteria were retained. 

In notable research by Liu and Cohen [13], carbonitride (CNx) coatings were found to exhibit hardness comparable to diamonds, particularly when involving covalent β-C_3_N_4_ bonding structures. Over the past 30 years, a few studies have been performed on CNx. Some results indicated that CNx coatings exhibited low friction, high adhesion, and high wear and corrosion resistance. Wäsche et al. found that among evaluated samples, a CNx coating showed the highest hardness under deposition conditions with a N/Ar ratio of 0.2 [14]. Takadoum et al. determined [15] that a CNx coating displayed higher wear resistance than a DLC coating. The same result has been obtained by Park et al. [16] and Camero et al. [17]. The CNx coatings, under deposition conditions with N/Ar ratios of 0.25–0.50, possessed the highest content of sp^3^ bonding and exhibited the highest wear resistance of their samples. 

Other studies have found that the properties of CNx coatings, such as hardness, corrosive resistance, antioxidation, and wear resistance, can be enhanced by metal doping [18,19,20,21]. 

Lai et al. [22] grew a series of CNx/amorphous C-Zr_0.55–0.60_ (a-C) coatings on biograde Ti samples. They found that the coatings showed high biocompatibility and good antibacterial performance when the C content was higher than 12.7 at. %. Huang et al. [23] prepared CNx coatings doped with Ti and Zr and evaluated the bacterial viability by using *S. aureus* and *Aggrigatebacter Actinomycetemcomitans*; the results revealed that bacterial viability levels of 21% and 36%, respectively, were obtained. Moreover, the coatings showed high biocompatibility with human fibroblasts. Considerably more studies have been conducted on mechanical properties than on antimicrobial performance.

Hosokawa et al. [24] prepared Ti-doped CNx (CNx-Ti) coatings on drills by using the magnetron sputtering method and conducting drilling tests. They revealed that the life of the CNx-Ti-coated drills was three times that of TiN-coated drills and that the flank wear decreased significantly as well. Kuptsov et al. [25] studied CNx-Ti coatings and found that corrosive resistance was improved by the formation of a surface TiO_2_ layer. The same mechanism was also found to achieve improvements in wear resistance at 300 °C [26]. 

In an identical study by Polcara et al. [26], Cr-doped CNx (CNx-Cr) coatings were prepared and evaluated using an elevated-temperature wear tester. The results showed that at 700 °C, the CNx-Cr coatings displayed a friction coefficient close to that at ambient temperature, due to minor variation in wear behavior between the two conditions. 

In a study on a series of Zr-doped CNx (CNx-Zr) coatings, the coatings with 34.3 at. % Zr and a C/N ratio of 0.9 showed a hardness of 22.8 ± 1.1 GPa, low friction, and high adhesion [27]. Wang et al. [28] found that deposition temperature influenced the properties of a CNx-Zr coating. As the deposition temperature increased, the mechanical properties improved. At a deposition temperature of 400 °C, the coating showed a hardness of 32.0 ± 0.3 GPa, good adhesion, and a favorable resilience coefficient. Grigore et al. [29] prepared CNx-Zr coatings using the magnetron sputtering method. Of the samples, the CNx-Zr coating showed the highest hardness (HV 3600) with a Zr content of 52 at. % under deposition of a N/Ar ratio of 0.1125, and that exhibited the highest wear resistance with a Zr content of 54.1 at. % under deposition of a N/Ar ratio of 0.0625. Yao et al. [30] prepared a series of CNx-Zr coatings with different N content levels and found that the coating with a Zr content of 48.7 at. % under deposition of a N/Ar ratio of 0.2 displayed the best wear resistance and highest hardness (HK 1600). 

Ospina et al. [31] evaluated the mechanical properties and wear performance of W-doped CNx (CNx-W) coatings prepared using the pulse arc deposition method. The results indicated that the critical load (*Lc*) and hardness increased, and the friction coefficient decreased to 0.35 from 0.7, as compared with a single W coating. Chen et al. [32] prepared CNx-W and WNx coatings and compared their wear behavior. The wear rates for the CNx-W and WNx coatings were 3 × 10^−8^ and 6 × 10^−8^ mm^3^/Nm, respectively. A large improvement in wear resistance was produced by the CNx-W coating.

Numerous studies have been conducted on DLC coatings involving various topics. Of these, only a few have discussed antibacterial activity and biocompatibility. Until now, according to our review of the literature, no data have been published concerning CNx coatings. In this study, we prepared CNx and 6 at. % metal-doped CNx coatings on SS316L substrates by using an industrial-scale four-target closed-field unbalanced DC magnetron sputtering system. The metals used were W, Ti, Zr, and Cr. The samples were characterized and evaluated for antibacterial and wear performance. This study was a trial of growing CNx and metal-doped CNx coatings on SS316L steel; their characteristics and possible applications against bacteria and against wear were investigated.

## 2. Experimental Details

### 2.1. Analysis Equipment

A scanning electron microscope (E-SEM Quanta 400F, FEI, Hillsboro, OR, USA) equipped with an electron dispersive X-ray system was used. A glow discharge spectrometer (GDS-750 QDP, LECO, Saint Joseph, MI, USA) was applied to determine the coating thickness. A glancing-angle X-ray diffractometer (D/MAX2500, Rigaku, Tokyo, Japan) was used, setting Cu Kα radiation at 40 kV and 100 mA with a glancing angle of 4°. An X-ray photoelectron spectroscope (PHI 1600 ESCA, Perkin-Elmer Co., Waltham, MA, USA) was used, employing non-monochromatized Mg Kα radiation. An X-ray photon spectroscopy (XPS) system was applied with 3 kV Ar ions to sputter the surface oxide layer and examine the chemical composition of the coatings. Spectra ranging from 0 to 1000 eV were recorded for each sample, followed by high-resolution spectra over different elemental peaks, from which the composition was calculated. The spectral ranges at 287 ± 7, 400 ± 9, 184 ± 10, 582 ± 13, 460 ± 10, and 36 ± 10 eV corresponded to the binding energies of C1s, N1s, Zr3d, Cr2p, Ti2p, and W4f, respectively. Curve fitting was performed after a Shirley background subtraction by a Gaussian fitting [33]. Energy calibration was conducted with reference to the Au 4f7/2 peak at 83.8 eV. A Raman spectrometer (HR 800 Micro PL, Horiba, Kyoto, Japan) employing a He-Ne laser and Peltier-cooled Princeton CCD camera was used. Two peaks were observed in the Raman analyses result, indicating carbon structures corresponding to a D peak at approximately 1363 cm^−1^ and a G peak at approximately 1560 cm^−1^. A nanoindentation system (LBI Nanoindenter, UNAT-M, Dresden, Germany) was used at an applied load of 10 mN. Adhesion was evaluated using a scratch tester with an applied load that increased from 0 to 100 N at a rate of 10 N/mm. A critical load (*Lc*) was obtained. 

### 2.2. Coatings

The coatings were prepared using a closed-field unbalanced DC magnetron sputtering system (CFUBMS KD-550U, MIRDC, Kaohsiung, Taiwan), which features four vertical orthogonally-mounted magnetron cathodes surrounding a rotational substrate holder. The dimensions of a target were 437.3 mm × 178.2 mm × 10.2 mm. 

Biograde SS316L was used as the substrate material. Two types of samples, block and plate, were used. The block had dimensions of 24 mm × 8 mm (diameter × thickness) and the plate of 16 mm × 16 mm × 1 mm. The plate sample was used in the antimicrobial test. The substrate surface to be evaluated was polished to a surface roughness Ra < 60 nm. 

The standard sample preparation procedure for physical vapor deposition (PVD) was used. After the samples were loaded, the chamber was pumped down to 2 × 10^−5^ Torr. The main Ar gas and minor H_2_ gas were introduced. The H ions were used to maintain the chamber temperature. The Ar ions were used to perform the bombardment conditioning (30 min). The Ar gas flow rate was increased at a steady rate until it reached 25 sccm (corresponding to 3 × 10^−3^ Torr) at the end of the bombardment process and was then maintained for the subsequent coating. 

In this study, CNx, CNx-W, CNx-Ti, CNx-Zr, and CNx-Cr coatings were prepared. For growth of metal-doped CNx coatings, four targets were used, with two carbon targets next to each other and two metal targets in the chamber. The metal targets were the doping material and were also used to prepare an effective interface. For example, in the case of the CNx-Ti coating, two Ti and two carbon targets were used. The Ti interlayer was prepared first under a condition of 1 A current for one Ti target for 10 min. The TiC gradual layer was then prepared under conditions of 1 A and 3 A currents for one Ti and two C targets, respectively, for 6 min. Finally, the main CNx-Ti coating was prepared under conditions of 1 A and 3 A currents for two Ti and two C targets, respectively, and a N_2_ gas flow rate of 6 sccm for 120 min. Identical processes were used to grow the CNx-Zr and CNx-Cr coatings by using Zr and Cr targets, respectively. The coating procedure for the CNx-W coating involved some modification. From long-term experience, we know that the use of a W interface layer leads to poor quality of adhesion for the subsequent main coating. Thus, to grow the CNx-W coating, two C targets, one Ti target, and one W target were used. The Ti interface was prepared first at a Ti target using a 1 A current for 10 min., and then the TiC gradual layer was prepared under conditions of 1 A and 2 A currents for one Ti and two C targets, respectively, for 6 min. Finally, the main CNx-W coating was prepared under conditions of 0.6 A and 3 A currents for one W and two C targets, respectively, and a N_2_ gas flow rate of 6 sccm for 120 min. The aforementioned conditions were set on the basis of repeated tests and trials for the different types of coatings. With these procedures, the overall thickness of the coatings was approximately 2 μm, as determined using glow discharge optical spectroscopy. The amount of doped metal was well controlled at 6–7 at. %, as determined using XPS. The data are listed in Table 1. For growth of the CNx coating, one Ti and two carbon targets were used. The Ti interface was prepared first, then the TiC gradual layer, and finally the main CNx coating, using the same condition for the CNx-Ti coating, with 0 A for the Ti target.

### 2.3. Hydrophilicity Test

The hydrophilicity of the surface of the samples was evaluated by measuring the static contact angles using a contact-angle analyzer (FTA-1000B, Portsmouth, VA, USA). Each specimen was alternately washed in ethanol and deionized water in an ultrasonic cleaner for 30 min and then dried at 55 °C for 4 h. A drop of 2 mL distilled water was place on the sample surface using a micrometric pipette at room temperature. The sample was immediately imaged, and the contact angle was measured automatically using a spherical fitting approach. Each contact angle reported herein is the mean of five independent measurements. 

### 2.4. Antimicrobial Test

The antimicrobial performance of a coating was evaluated according to the antimicrobial activity of a given coating surface against bacteria in contact with it. The antimicrobial test procedures were performed following the Japan Industrial Standard JIS Z 2801:2010 Antimicrobial products—Test for antimicrobial activity and efficacy, with minor modifications. Prior to the test, the plate samples were sterilized in a high-pressure steam autoclave operated at 121 °C, 103 kPa for 15 min, and then placed in sterile Petri dishes. The test bacteria used were *S. aureus* Gram-positive bacteria in suspension, adjusted at a cell concentration of 5 × 10^8^ colony-forming units per milliliter (cfu/mL). A 50 μL suspension of the test bacteria was instilled onto the surface of a sample, and was then covered with a sterilized polyethylene film measuring 10 mm × 10 mm. The film was pressed so that the inoculum (test bacteria) spread evenly over the film, to avoid drop evaporation. An uncoated SS316L sample was used as a reference. Finally, the Petri dishes containing the inoculated test pieces were incubated at a temperature of 37 °C and a relative humidity of not less than 90% for 24 h.

After the inoculation, the test bacteria were washed out using 10 mL of phosphate-buffered saline. To conduct a viable cell count of bacteria, 10-fold serial dilutions and the agar plate culture method were used. Commercial Lysogeny broth agar was used (Difco Laboratories, Detroit, MI, USA). The incubation was performed at 37 °C for 24 h. After incubation, the number of visible bacterial colonies was counted using an agar plate in which 30 to 300 colonies appeared. To determine the antimicrobial performance, the following formula was used:

*Antibacterial Efficiency* (*η*) = (N_0_ − N)/N_0_ × 100%

where N (expressed in cfu) represents the number of viable cells of bacteria on the sample and N_0_ represents the number for the uncoated SS316L reference sample. The N value was calculated using C*D, where C is an average of the number of colonies in three test pieces, and D is the corresponding dilution ratio of the diluted solution. The data presented herein were obtained from seven independent experiments.

The final condition of the bacterial colonies was imaged using a traditional camera. The statistical correlation of the antibacterial efficiency between the coated samples was determined using Student’s *t*-test [34]. Differences were considered to be statistically significant when *p* was less than 0.01.

### 2.5. Wear Test 

The wear performance of the coatings was evaluated using a pin-on-disk rotating-sliding wear tester (Jun-Yan Precision Machine Co., Kaoshiung, Taiwan). The material of ball attached at the pin end was 100% Al_2_O_3_. The testing conditions were as follows: applied load, 10 N; line speed, 0.2 m/s; rotating radius, 6 mm; and sliding distance, 350 m. The resulting wear track was measured by scanning four cross-sections at an interval of 90° using a white-light interferometer (BMT, Nuremberg, Germany). The wear rate was calculated by the formula 2πRA/WL, where R is the rotating radius, A is an average of four measurements of a wear cross area, W is the load, and L is the sliding distance. 

## 3. Results and Discussion

### 3.1. Coatings

**Figure 1:**
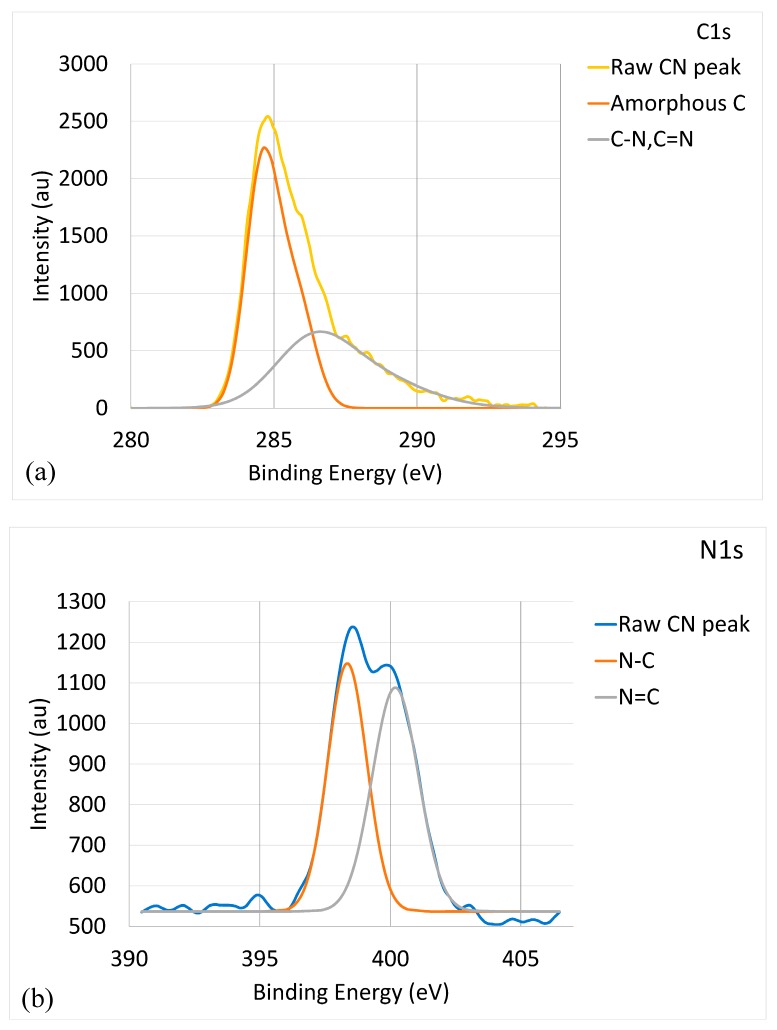
Bonding energy of CNx coating characterized by XPS: (**a**) C1s XPS raw and deconvoluted spectra; and (**b**) N1s XPS raw and deconvoluted spectra.

Figure 1 shows the binding energy of the CNx coating characterized by XPS. Figure 1a provides the C1s core-level XPS spectrum. A major peak was observed at 284.8 eV, with a minor protrusion at 285.9 eV, which could be deconvoluted into two components with the broader peak corresponding to (C, N) bonding and the other peak corresponding to a-C. The eV values of C-N (286.3 eV) and C = N (285.5 eV) peaks are close and mostly overlap each other [35,36]. The deconvolution of the C-N/C = N peak is un-meaningful [37]. It can be concluded that the (C, N) peak was composed of C-N and C = N peaks. The spectrum of a-C phase at 284.5 eV covered a wide range of 283–287 eV, suggesting the formation of sp^2^ a-C (284.2 eV) [38] and sp^3^ a-C (284.9 eV) [39]. The presence of a-C structures in the CNx coating was further confirmed by the Raman spectroscopy analysis results, as listed in Table 2. Raman spectroscopy is a highly effective method for characterizing the detailed bonding structure of carbon nanomaterials. When the ID/IG value is >1, a-C is present, including sp^2^ a-C and sp^3^ a-C. With a higher ID/IG value, the material is more amorphous. The data in Table 2 indicate that metal doping led to an increase in amorphization. For the CNx coating, the ID/IG value was 1.49, which indicates the presence of a-C. Figure 1b shows the N1s core-level XPS spectrum. The raw CNx peak could be deconvoluted into two components corresponding to a N-C peak at 398.3 eV and a N = C peak at 400.2 eV [40]. Figure 1b and the ID/IG value indicate that the CNx coating was a mixture of CNx and a-C phases. 

**Figure 2:**
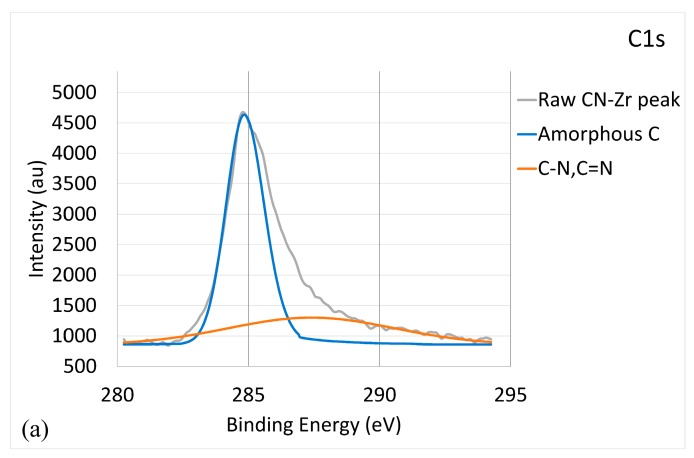

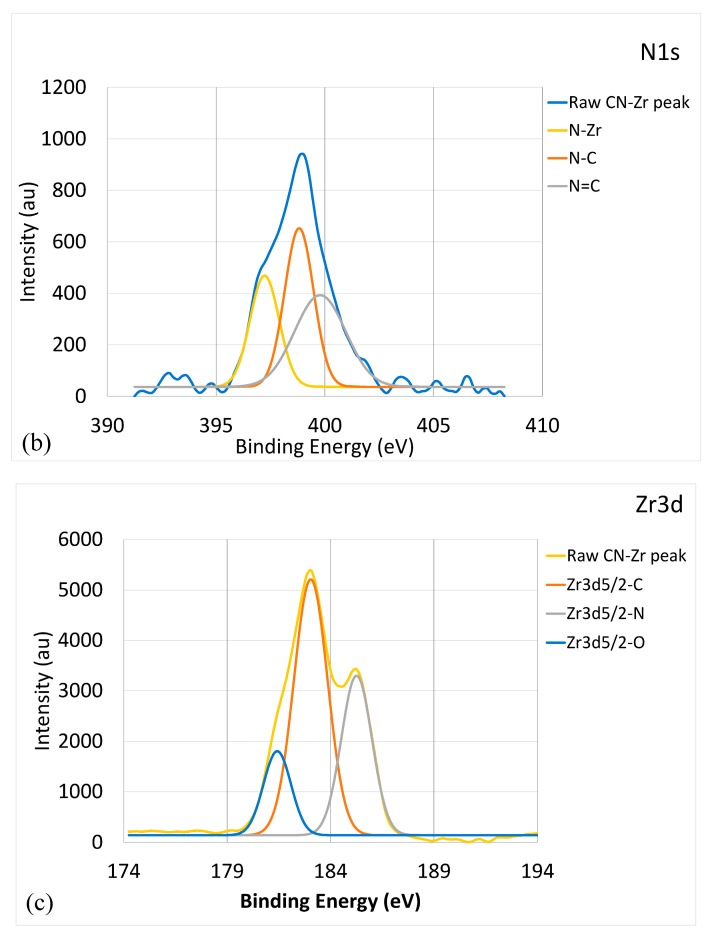
Bonding energy of CNx-Zr coating characterized by XPS: (**a**) C1s XPS raw and deconvoluted spectra; (**b**) N1s XPS raw and deconvoluted spectra; and (**c**) Zr3d raw and deconvoluted spectra.

Figure 2 illustrates the bonding energy of CNx-Zr coating characterized by XPS. Figure 2a provides the C1s core-level XPS spectrum. The deconvolution produced similar results to Figure 1a, but with a lower strength for the (C, N) peak. The C-Zr peak was very weak and could not be distinguished with certainty. The ID/IG value was 2.85, indicating the presence of a-C. Figure 2b shows the N1s core-level XPS spectrum. The broad peak could be deconvoluted into three components corresponding to a major N-C peak at 398.2 eV, a N = C peak at 400.1 eV, and a N-Zr peak at 397.1 eV [40,41]. Figure 2c presents the Zr3d core-level XPS spectrum. The deconvolution indicated a major Zr-C peak at 182.9 eV, a Zr-N peak at 185.1 eV, and a Zr-O peak at 181.5 eV [42]. The presence of Zr-O was caused by the improper storage of the sample prior to performing the XPS analyses. Therefore, the result reveals that the CNx-Zr coating comprised a-C, CNx, and ZrC/ZrN phases. 

**Figure 3:**
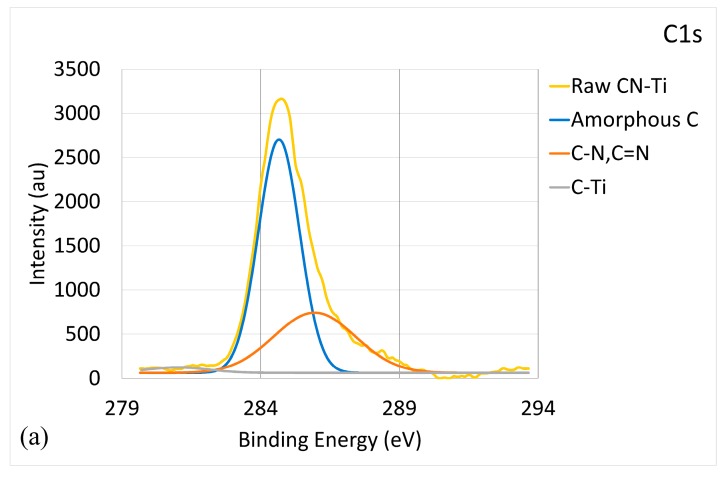

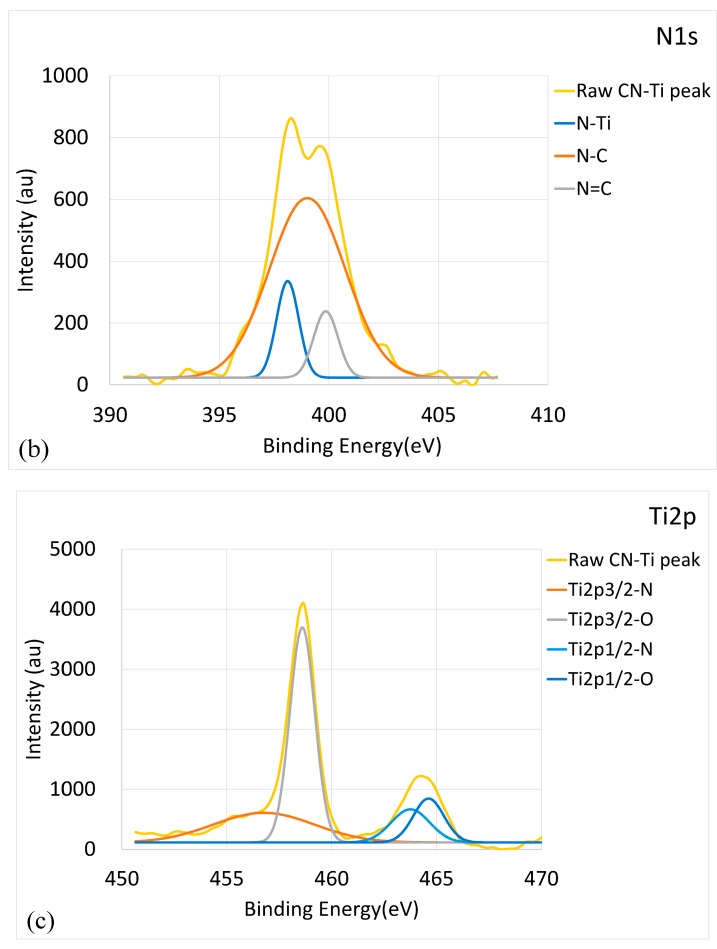
Bonding energy of CNx-Ti coating characterized by XPS: (**a**) C1s XPS raw and deconvoluted spectra; (**b**) N1s XPS raw and the deconvoluted spectra; and (**c**) Ti2p raw and deconvoluted spectra.

The analyses of XPS spectra of the CNx-W coating showed that it was a mixture of a-C, CNx, and WC/WN phases. Similarly, the CNx-Cr coating consisted of a-C, CNx, and CrC/CrN phases. However, the CNx-Ti coating differed, as illustrated in Figure 3. Figure 3a shows the C1s core-level XPS spectrum. The deconvoluted peaks included an a-C peak and a C-N peak; the C-Ti peak (281.7 eV) [43] nearly approached zero. This indicates that TiC was present in minute amounts, or absent. As shown in Figure 3b, the deconvolution of the N1s core-level XPS spectrum included a major N-C peak, a N = C peak, and a N-Ti peak (397 eV) [41]. Figure 3c provides the Ti2p core-level XPS spectrum, in which, after deconvolution, two Ti-N peaks (455.8 and 460.9 eV [44]) and two Ti-O peaks (454.6 and 464.7 eV [45]) could be obtained. Improper storage of the sample before performing the XPS analyses resulted in the appearance of Ti-O. No Ti-C bonding was detected. Therefore, the CNx-Ti coating was a mixture of a-C, CNx, and TiN phases; no TiC phase was grown. 

**Figure 4:**
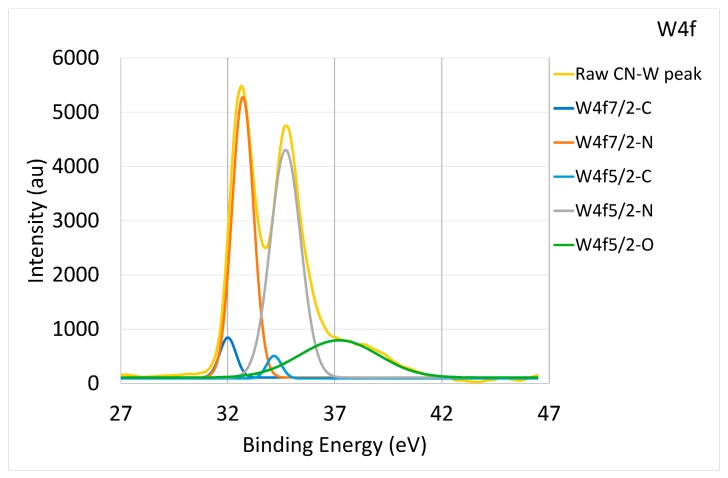
W4f XPS raw and deconvoluted spectra derived from bonding energy of CNx-W coating characterized by XPS.

When the bonding concentration of C and N was derived from the doped metals’ core-level XPS spectrum, the binding states of carbide and nitride could be compared. As presented in Figure 2c, the content of Zr-C bonding was higher than that of Zr-N; the same was true for Cr-C and Cr-N. Comparatively, as shown in Figure 4, the content of W-N bonding was higher than that of W-C bonding in the CNx-W coating. These results demonstrate the different tendencies of each metal to form C or N compounds.

**Figure 5:**
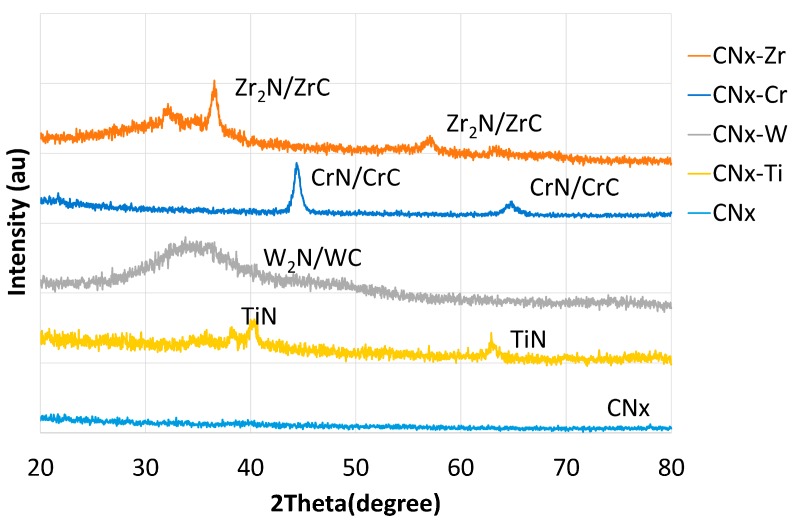
XRD patterns of CNx and 6 at. % metal-doped CNx coatings.

Typical X-ray diffraction (XRD) spectra for the CNx and 6 at. % metal-doped CNx coatings are shown in Figure 5. For the CNx coating, no obvious crystalline peak was evident, indicating the presence of an amorphous CNx (a-CNx) structure. Thus, the results of XRD, XPS (Figure 1), and Raman analyses (Table 2) indicated that the CNx coating was a composite containing a-CNx and a-C structures. Under the applied conditions, pure a-CNx could not be obtained. For simplicity, the term “CNx” is used in the following. For the other metal-doped coatings, crystalline phases were evident. For the CNx-Ti coating, the results of XRD, XPS, and Raman analyses indicated that the CNx coating functioned as a matrix, with the crystalline TiN phase dispersed in it. The lack of formation of the TiC phase was also confirmed. For the other doped CNx coatings, different types of carbides and nitrides were present. The results of XRD, XPS, and Raman analyses indicated that the CNx-W coating was a composite comprising crystalline W_2_N and WC phases dispersed within the CNx coating. The CNx-Cr and CNx-Zr coatings had crystalline CrN/CrC phases and Zr_2_N/ZrC phases, respectively, which were dispersed within the CNx coating. 

**Figure 6:**
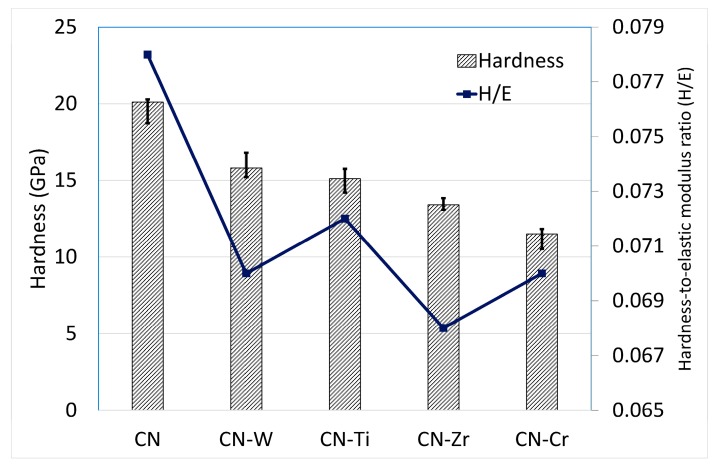
Hardness and hardness-to-elastic modulus ratio (H/E) values of CNx and 6 at. % metal-doped CNx coatings.

Figure 6 provides a comparison of the coatings with respect to their hardness and hardness-to-elastic modulus ratio (H/E). The CNx coating exhibited the highest values. Metal doping reduced the hardness values. These results indicate that the metal-doped CNx coatings consisted of a CNx coating with carbide/nitride compound(s) dispersed throughout. These compound(s) impaired the overall integrity of the coatings. Thus, the hardness of the metal-doped CNx coatings was lower than that of the CNx coating. Moreover, the different types of nitride/carbide compounds formed and their dispersed states and the a-CNx/a-C ratio also affected the hardness. The hardness of the metal-doped coatings, ranked in descending order, was CNx-W > CNx-Ti > CNx-Zr > CNx-Cr. When a nanohardness tester is used, the Young’s modulus can be measured simultaneously. The H/E values were calculated, and their order, ranked in descending order, was CNx-Ti > CNx-W > CNx-Cr > CNx-Zr. 

The average surface roughness (Ra) of the CNx coating was very low, at 31 nm (Table 2). Metal doping caused the Ra values to be reduced by 50–70%, as a result of the increase in amorphization. All the coatings exhibited comparable adhesion values, ranging from 68 N to 80 N. 

### 3.2. Hydrophilicity Test

The hydrophilicity of a surface is an important factor when studying the viability of bacteria. Typically, the higher the hydrophilicity (lower wettability) of a surface is, the higher the contact angle and its antibacterial performance are [46]. Hydrophilicity is influenced by surface properties such as chemical characteristics and morphology. 

**Figure 7:**
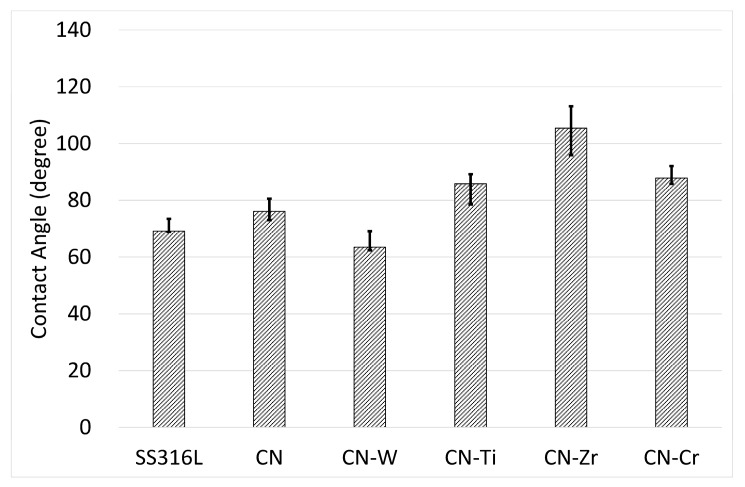
Contact angle values of uncoated SS316L and CNx- and 6 at. % metal-doped CNx-coated samples.

The results of the hydrophilicity test for the uncoated and coated samples are illustrated in Figure 7. The CNx coating exhibited a slightly higher angle than the uncoated SS316L sample. The CNx-Ti, CNx-Zr, and CNx-Cr coatings displayed further increases in their contact angles. However, the angle of the CNx-W coating was lower than that of the uncoated SS316L sample, despite the CNx-W coating having very low surface roughness (Table 2). The CNx-Zr coating, which had a medium-level Ra value, exhibited the highest contact angle. Thus, for these coated samples, the surface morphology was not the main factor affecting the contact angle. Chemical characteristics may be the main cause because each coating had a different composition and constituent phases.

### 3.3. Antimicrobial Test

*S. aureus* and *A. actinomycetemcomitans* are considered common causes of postoperative infection and inflammatory reactions [47,48], which begin with bacterial colonization of implants or tissue surfaces. Biomaterials and medical tools with antibacterial surfaces may help to reduce the formation of bacterial colonies and thereby avoid inflammation. 

**Figure 8:**
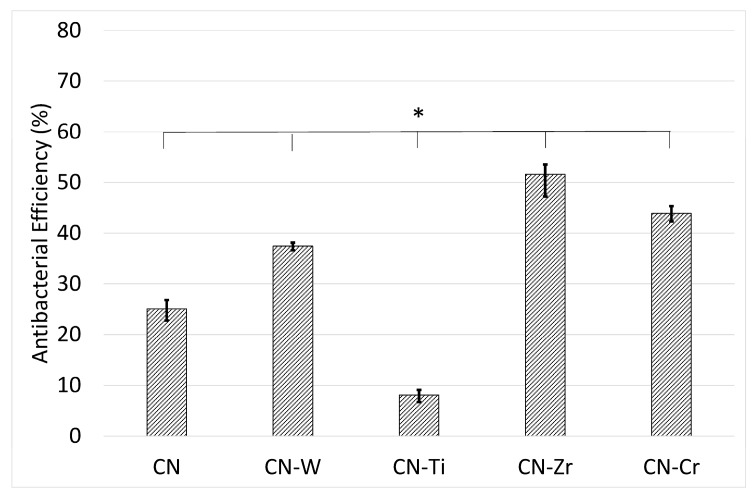
*Antibacterial efficiency* (*η*) evaluated using *Staphylococcus aureus* on CNx- and 6 at. % metal-doped CNx-coated SS316L samples, with the uncoated SS316L ones serving as a reference. (*) A value of *p* < 0.01 was considered to indicate a significant difference between the values of the two groups of samples.

In the present study, *S. aureus* was incubated on the surfaces of uncoated SS316L and CNx- and 6 at. % metal-doped CNx-coated plates to evaluate their antimicrobial performance. By applying the uncoated SS316L sample as a reference, we could obtain the *antibacterial efficiency* (*η*) values (Figure 8). The results revealed that *η* = 25% for the CNx coating, indicating that the CNx coating reduced the bacterial count of inoculum by 25% relative to the uncoated SS316L plate. These experimental results indicate that all the coatings showed antibacterial ability. The CNx-Zr coating displayed the highest antimicrobial performance and had a substantially higher *η* value than the single CNx coating. Second most effective was the CNx-Cr coating, followed by the CNx-W coating. However, the CNx-Ti coating exhibited markedly lower antibacterial performance than the CNx coating. 

**Figure 9:**
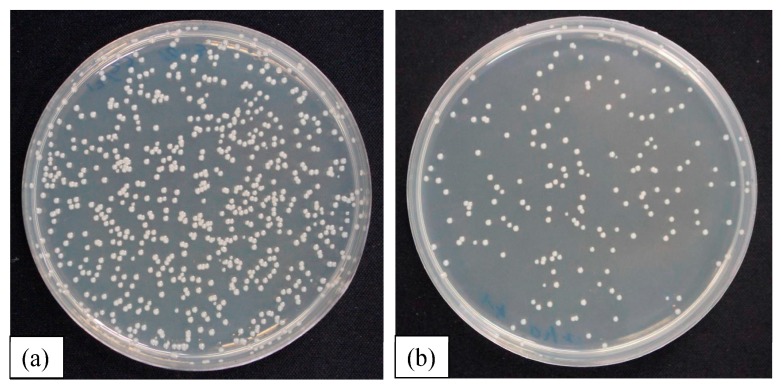
Typical photographs of visualized colonies on the agar plates after incubation for 24 h on (**a**) uncoated SS316L; and (**b**) CNx-Zr coated samples.

Figure 9 depicts typical photographs of bacterial colonies on the agar plates after incubation for 24 h on the uncoated SS316L (Figure 9a) and CNx-Zr coated (Figure 9b) samples. The antimicrobial performance of the CNx-Zr coatings was very pronounced. These results confirm that the numbers of viable bacteria were lower on the CNx and 6 at. % metal-doped CNx coatings than on the uncoated SS316L reference sample. 

The antibacterial ability of the coatings appeared to be related to hydrophilicity. The CNx-Zr and CNx-Cr coatings, which exhibited high hydrophilicity, displayed excellent antibacterial performance. However, this relation did not apply to the other coatings. The CNx-Ti coating, which had a highly hydrophilic surface, showed the lowest antibacterial ability. Some researchers reported that C aggregates combat microorganisms by physically damaging the outer membranes of cells [49]. This can explain the increase in antibacterial performance of the coatings in the current study, because all the coatings were carbon-based. However, the coatings also contained N, and N-C bonding was detected. In addition, the coatings contained metal carbides/nitrides. To date, no related data have been reported. Based on these interaction effects, the causes of the different antimicrobial performance levels are complex and need further investigation. 

### 3.4. Wear Test

**Figure 10:**
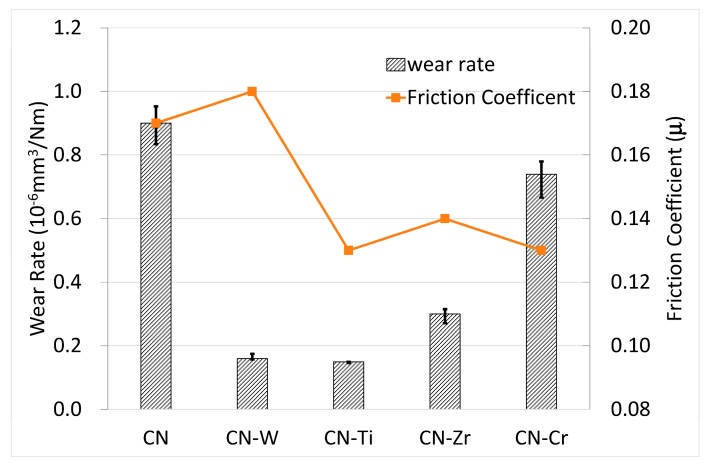
Comparison of wear test results of CNx and 6 at. % metal-doped CNx coated samples

The results of the wear test are illustrated in Figure 10. The data for the uncoated SS316L sample are not included due to its very high value, which was twice that of the CNx coating. The CNx coating showed the lowest wear resistance, whereas metal doping greatly improved wear resistance. The CNx-W and CNx-Ti coatings displayed the highest wear resistance and were comparable to each other. Second was the CNx-Zr coating. Finally, the CNx-Cr coating exhibited the lowest wear resistance. Regarding the detected friction coefficient, there was no direct relationship with wear behavior. 

Figure 6 presents the H/E values. A rule proposed by Affonso [50] is commonly used to predict the wear resistance of materials, either in bulk or coating form, by using H/E values. The higher the H/E value is, the higher the wear resistance. Here, this rule cannot be effectively applied. By comparing the wear rate in Figure 10 and coating hardness in Figure 6, we could obtain a relationship for the four metal-doped coatings. The wear resistance followed the same trend as that of the hardness values. That is, coatings with higher hardness showed lower wear rates. Therefore, the wear resistance of the materials had the order CNx-W ≒ CNx-Ti > CNx-Zr > CNx-Cr, following the trend of the hardness values. 

## 4. Conclusions

SS316L materials have been widely applied in the field of biomaterials and medical tools. However, it shows relatively low wear resistance. In this study, we prepared CNx and 6 at. % metal-doped CNx coatings on SS316L substrates by using an industrial-scale four-target closed-field unbalanced DC magnetron sputtering system. The metals used were W, Ti, Zr, and Cr. The coatings were characterized and studied for antimicrobial and wear performance. Under the applied conditions, the CNx coating was found to be a composite containing a-CNx and a-C structures. For the metal-doped coatings, the metal reacted with C/N to form crystalline carbides/nitrides that were dispersed in the CNx coatings. Metal doping reduced the hardness and surface roughness. No substantial difference in adhesion was observed between coatings. All the coatings, both CNx and metal-doped CNx, exhibited antimicrobial ability relative to the uncoated SS316L sample. Of the doped coatings, the CNx-Zr coating showed low surface roughness and the highest hydrophilicity. The CNx-Zr coated sample displayed excellent antibacterial performance, despite having slightly lower wear performance. The favorable results for the CNx-Zr coating are very encouraging due to the high biocompatibility of C, N, and Zr elements. The CNx-Zr coating merits further study.

## Figures and Tables

**Table 1:** Composition and thickness of CNx and 6 at. % metal-doped CNx coatings.

Samples	Composition (at. %)			Coating Thickness (μm)
	Me	C	N	
CNx	-	68	32	2.0
CNx-W	6	60	34	2.1
CNx-Ti	6	58	36	1.6
CNx-Zr	7	62	31	1.8
CNx-Cr	6	58	36	1.9

**Table 2:** Surface roughness, adhesion and ID/IG values (Raman analyses) of CNx and 6 at. % metal-doped CNx coatings.

Samples	Surface Roughness Ra (nm)	Adhesion *Lc* (N)	ID/IG
CNx	31	68	1.49
CNx-W	12	68	1.63
CNx-Ti	10	74	2.10
CNx-Zr	17	80	2.85
CNx-Cr	18	75	1.72
